# Acute Adult Supraglottitis: An Impending Threat to Patency of Airway and Life

**DOI:** 10.7759/cureus.9976

**Published:** 2020-08-23

**Authors:** Nissar Shaikh, Shoaib Nawaz, Kiran Ahmad, Muna Al Maslamani

**Affiliations:** 1 Anesthesia, Hamad Medical Corporation, Doha, QAT; 2 Infectious Diseases, Hamad Medical Corporation, Doha, QAT

**Keywords:** apnea, dysphagia, dysphonia, ludwig’s angina, sore throat, streptococcal species, tracheostomy, adult acute supraglottitis (aas)

## Abstract

Introduction

Acute adult supraglottitis (AAS) is one of the upper airway infections that can potentially cause upper airway obstruction and, if not treated promptly, can be life-threatening. The widespread use of vaccines against *Hemophilus influenzae* has decreased the incidence of epiglottitis in children, whereas the incidence of AAS is on the rise. We aim to highlight the presentation, diagnosis, and management in AAS with our study.

Patients and Methods

A retrospective analysis was performed on all patients admitted to a tertiary health care facility surgical intensive care unit (SICU) where AAS was identified and the demographic data, duration of symptoms, imaging studies, management, and complications were recorded. In these patients, the diagnosis of AAS was confirmed by nasopharyngeal endoscopy. Data was entered in the IBM Statistical Package for Social Sciences (SPSS), version 23 (IBM SPSS Statistics, Armonk, NY), and groups were compared using student t-test and chi-square test. P values of ≤ 0.05 were considered statistically significant.

Results

A total of 118 patients were admitted to the SICU. The male: female ratio was 3.9: 1. Major risk factors were smoking and drinking cold liquids. The common presenting symptom was sore throat (89.8%). The thumb sign was positive in 65% of the patients. Common bacteria were the *Streptococcus* species (11.9%). Ceftriaxone was the most commonly prescribed inpatient antibiotic. All patients received steroids as adjuvant therapy. Adrenaline nebulization was used in 66% of the cases. Forty-six percent of patients required endotracheal intubation. In 10.2% of patients, intubation was not possible and in 12.7% of patients, a tracheostomy was done. Ludwig’s angina was the most frequent complication. Patients presenting with dysphagia and fever had a significantly higher incidence of Ludwig’s angina (P ≤ 0.02 and 0.005, respectively). AAS patients complicating into Ludwig’s angina (severe cellulitis of submandibular, submental, and sublingual spaces) had a significantly longer duration of symptoms, a higher incidence of streptococcal infection, airway interventions, and prolonged stay in an intensive care unit (p ≤ 0.05).

Conclusion

Male gender, smoking, and drinking cold liquids were the risk factors associated with AAS, and thumb sign on lateral neck soft tissue x-ray was suggestive of it. AAS caused by *Streptococcus* species was a relatively serious condition, leading to complications like Ludwig’s angina.

## Introduction

Acute adult supraglottitis (AAS) used to be called epiglottitis in the past. Supraglottitis is the more appropriate terminology in adults in contrast to the epiglottitis in children where the infection commonly involves only epiglottis. AAS is not only infection and inflammation of epiglottis but also of the surrounding structures namely arytenoid, uvula, the false vocal cords, and laryngeal ventricles [1-2]. Acute adult supraglottitis (AAS) is a serious and potentially life-threatening disease causing airway obstruction which, if not diagnosed early and treated promptly, can lead to high morbidity and mortality. [1] In the past, bacterial epiglottitis was common in children and was caused by Haemophilus influenzae type b (Hib). By the strict implementation of nationalized vaccine programs against H. influenzae in children, the rate of epiglottitis decreased in children from 99 to one case in 100,000. At the same time, the incidence of supraglottitis in the adult population increased from 0.88 to 3.1/100,000 patients [2]. An even higher incidence (4.7/100,000) of AAS was reported in Finland [3]. The literature from this region about AAS is not available, and as per most international studies, the majority of the patients, including milder cases of supraglottitis, are managed in the intensive care setup.

## Materials and methods

After obtaining permission from the Institutional Medical Research Committee, Hamad Medical Corporation, Doha, all patients admitted to the surgical intensive care unit (SICU) of the only tertiary health care centre and university hospital during the period from January 2008 to December 2018 were identified from the register and included in the study. The demographic data, comorbidities, history of smoking, tobacco chewing, the recent history of ice-cold fluid intake (cold drinks/ice cream/etc.), duration of symptoms, initial signs and symptoms upon presentation, imaging studies, microbiology, antibiotic therapy, airway interventions and management, length of ICU (intensive care unit) stay, complications, and outcomes of all patients were recorded.

In all patients, the diagnosis of supraglottitis was confirmed by fiberoptic nasopharyngeal endoscopy (performed by the otolaryngology on-call specialist) which showed swelling, edema, inflammation, and infection of the epiglottis and surrounding structures. Exclusion criteria were all paediatric patients, adult patients who sustained trauma, patients who ingested corrosive liquids, and patients with radiotherapy-induced supraglottitis.

In our patient population, dysphagia was defined as difficulty in swallowing, dysphonia as a change in speech tone (muffled and/or hoarseness), sore throat as severe pain in the throat, stridor as noisy inspiration, and dyspnea as difficulty in breathing. Fever was defined as a temperature of more than 38.3° C (taken either from the oral cavity or forehead infrared scan) and leucocytosis when the white blood cell (WBC) count was more than 11 x 10^3^ cells/µL. A throat swab was taken for microbiological evaluations. Others in microbiology included *Escherichia coli* (E. coli), anaerobes, *Acinetobacter*, and methicillin-resistant *Staphylococcus* aureus (MRSA).

Data were entered and analysed using the IBM Statistical Package for Social Sciences (SPSS), version 23 (IBM SPSS Statistics, Armonk, NY). Categorical variables were reported using numbers (n) and percentages (%). Continuous variables were reported as mean ± standard deviation (SD). The Kolmogorov-Smirnov test proved these variables to be normally distributed. Comparisons between groups were performed by using the chi-square test for categorical variables and the Student t-test for continuous variables. A two-tailed p-value of ≤ 0.05 was considered statistically significant.

## Results

During the study period, a total of 118 patients were admitted to the SICU with acute adult supraglottitis (AAS). The majority of our patients were male (94/79.7%) with a male: female ratio of 3.9: 1 (Table 1). The majority of comorbid conditions in our patients included diabetes mellitus in 35 (29.7%) and hypertension in 34 (28.8%) patients. Fifty-one patients (43.2%) had a history of smoking and 49 patients (41.5%) had a recent history of drinking ice-cold liquids, including drinks with added ice cubes and ice cream. The most common presentation of AAS was sore throat in 106 patients (89.8%), followed by fever in 103 patients (87.3%), dysphonia in 103 patients (87.3%), stridor in 102 (86.4%), dysphagia in 97 (82.2%), and dyspnea in 69 patients (58.5%). Leucocytosis was present in the majority of AAS patients (103 patients, 87.3%) (Table 1). The thumb sign on lateral neck soft tissue x-ray was positive in the majority of patients (77 patients, 65.2%), although an x-ray was not done in 25 patients (21.2%) (Table 2). Computerized tomography (CT) scan of the neck was performed in 53 patients (44.9%) in which the diagnosis was not clear on presentation. The CT scan was done within 24 hours of presentation. A microbiological evaluation was not done in the majority of patients (87 patients, 73.1%). Common microbial growth was streptococcal (14 patients, 11.9%), followed by *H. influenzae *(five patients, 4.2%), *Klebsiella pneumoniae *(*K. pneumoniae*) (five patients, 4.2%), *Candida albicans* (three patients, 2.5%), and others (four patients, 3.3%). The commonly used antimicrobial agent was ceftriaxone, followed by piperacillin + tazobactam in 20 (16.9%), amoxicillin + clavulanic acid in 12 (10.2%), meropenem in six (5.1%), azithromycin in five (4.2%), and antifungals (anidulafungin) in three patients (2.5%). Adrenaline nebulization was used in 78 patients (66.1%). Steroids were used in all of our patients. Dexamethasone was the most commonly prescribed steroid that was used in 100 patients (84.7%). Other steroids prescribed were hydrocortisone (16 patients, 13.6%) and methylprednisolone (two patients, 1.7%). Sixty patients (50%) did not require any airway interventions, 46 patients (39%) required intubation using video laryngoscopy or fibrotic bronchoscopy (as they were difficult to intubate), and in 12 patients (10.2%), intubation was not possible. Tracheostomy was required in 15 patients (12.7%) (Table 2). There was no mortality in our case series.

**Table 1:** Descriptive Data

Variable		Number	Percentage
Gender	Female	24	20.3
	Male	94	79.7
HTN (hypertension)	Yes	34	28.8
	No	84	71.2
DM (diabetes mellitus)	Yes	35	29.7
	No	83	70.3
Smoking	Yes	51	43.2
	No	67	56.8
Cold drinks	Yes	49	41.5
	No	69	58.5
Sore throat	Yes	106	89.8
	No	12	10.2
Dysphonia	Yes	103	87.3
	No	15	12.7
Fever	Yes	103	87.3
	No	15	12.7
Leucocytosis	Yes	103	87.3
	No	15	12.7
Stridor	Yes	102	86.4
	No	16	13.6
Dysphagia	Yes	97	82.2
	No	21	17.8
Dyspnea	Yes	69	58.5
	No	49	41.5

**Table 2:** Imaging Findings, Microbiology, and Treatment of Acute Adult Supraglottitis (AAS) MRSA: Methicillin-resistant *Staphylococcus aureus*

Variables		Number	Percentage
Thumb Sign	Positive	77	65.2
	Negative	16	13.6
	X-ray not done	25	21.2
Computed Tomography (CT) Neck	Yes	53	44.9
	No	65	55.1
Microbiology	Not done	87	73.1
	Streptococcus species	14	11.9
	H. influenzae	5	4.2
	K. pneumoniae	5	4.2
	Candida species	3	2.5
	Others (E. coli, MRSA, Anaerobes, Acinetobacter)	4	3.3
Antimicrobial Agents	Ceftriaxone	72	61
	Piperacillin + Tazobactam	20	16.9
	Amoxicillin + Clavulanic Acid	12	10.2
	Meropenem	6	5.1
	Azithromycin	5	4.2
	Antifungal	3	2.5
Adrenaline Nebulization	Yes	78	66.1
	No	40	33.9
Steroids	Dexamethasone	100	84.7
	Hydrocortisone	16	13.6
	Methylprednisolone	2	1.7
Endotracheal Intubation	Not intubated	60	50.8
	Not possible	12	10.2
	Intubated	46	49
Tracheostomy	Yes	15	12.7
	No	103	87.3

Table 3 shows the patients’ age, duration of symptoms, and complications. The mean age of the patients was 43.91 years. The mean duration of symptoms and length of ICU stay were 2.92 and 6.59 days, respectively. In 47 patients (38.8%), there were no complications. The common complication in our AAS population was the development of multiple abscesses or Ludwig's angina (bilateral spread of infection to the submandibular space and the sublingual space forming abscesses). However, the possibility of an odontogenic infection leading to swelling of the glottic area could not be ruled out. Twenty patients (16.9%) had obstruction to the upper airway, leading to apnea (Table 3).

**Table 3:** Patient Age, Durations, and Complications

Variables	Mean ± SD (Standard Deviation)	
Age (years)	43.91 ± 17.37	
Duration of symptoms (days)	2.92 ± 1.85	
Intensive care unit stay (days)	6.59 ± 5.48	
Complications	Number	Percentage
No complications	47	38.8
Ludwig’s Angina	33	28.0
Apnoea	20	16.9

Table 4 shows variables associated with the complications of AAS leading to Ludwig’s angina. The presence of fever and dyspnea are significantly associated with a prediction for Ludwig’s angina (p = 0.001 and 0.005, respectively). Patients with streptococcal infection had a significantly increased number of complications including Ludwig's angina (p = 0.001). AAS patients complicated by Ludwig’s angina required a significant increase in airway interventions, both intubations and tracheostomies (p = 0.001 and 0.006, respectively). Those patients had a longer duration of symptoms and a higher length of intensive care stay (p = 0.001).

**Table 4:** Variables Associated With Ludwig’s Angina MRSA: Methicillin-resistant *Staphylococcus aureus*

Variables		Ludwig’s Angina		P-value
		No (Number & Percentage)	Yes (Number & Percentage)	
Gender	Female	17 (20.0)	7 (21.2)	0.54
	Male	68 (80.0)	26 (78.8)	
Hypertension	No	64 (75.3)	20 (60.0)	0.08
	Yes	21 (24.7)	13 (39.4)	
Diabetes mellitus	No	61 (71.8)	22 (66.7)	0.37
	Yes	24 (28.2)	11 (33.3)	
Smoking	No	51 (60.0)	16 (33.3)	0.17
	Yes	34 (40.0)	16 (48.5)	
Cold drinks	No	53 (62.4)	16 (48.5)	0.17
	Yes	32 (37.6)	17 (51.5)	
Dysphagia	No	19 (22.4)	2 (6.1)	0.02
	Yes	66 (77.6)	31 (93.9)	
Dysphonia	No	13 (15.3)	2 (6.1)	0.14
	Yes	72 (84.7)	31 (93.1)	
Sore throat	No	11 (12.9)	1 (3.0)	0.09
	Yes	74 (87.1)	32 (97.0)	
Dyspnea	No	43 (50.6)	6 (18.2)	0.001*
	Yes	42 (49.4)	27 (81.8)	
Fever	No	15 (17.6)	0 (0)	0.005*
	Yes	70 (82.4)	33 (100)	
Leucocytosis	No	13 (15.3)	2 (6.1)	0.14
	Yes	72 (84.7)	31 (93.9)	
Bacteria (Culture)	Not done	74 (87.1)	13 (39.4)	0.001*
	Streptococcus	3 (3.5)	11 (33.3)	
	H. influenzae	3 (3.5)	2 (6.1)	
	K. pneumoniae	2 (2.4)	3 (9.1)	
	Fungus	0 (0)	3 (9.1)	
	Others (E. coli, anaerobes, MRSA, Acinetobacter)	3 (9.1)	2 (6.1)	
Intubation	Not Intubated	59 (69.4)	1 (1.7)	0.001*
	Not possible	4 (4.7)	8 (24.2)	
	Intubated	22(25.9)	24 (72.8)	
Tracheostomy	No	79 (92.9)	24 (72.7)	0.006*
	Yes	6 (7.1)	9 (27.3)	
Variables		Mean ± SD		p-value
Age (years)	No	43.59 ± 18.43		0.13
	Yes	44.73 ± 14.52		
Duration of symptoms (days)	No	2.52 ± 1.7		0.001*
	Yes	3.94 ± 1.6		
Intensive care unit stay (days)	No	5.14 ± 4.06		0.001*
	Yes	10.33 ± 6.82		

## Discussion

AAS is a potentially life-threatening infection due to the possibility of upper airway obstruction. Hence, these patients are preferably monitored in the intensive care environment [1-3]. AAS is a severe inflammation and infection of the epiglottis and surrounding structures. These structures have loose mucosa and a good blood supply; thus, the infection spreads quickly with a high risk of sudden fatal upper airway obstruction [3]. Ng et al. found that the AAS was more common in males with an M: F (male: female) ratio of 3: 1 [4]. Bizaki et al., however, reported a lower M: F ratio of 1.6: 1 in their review of 308 patients [3]. In our patient population, the M: F ratio was 3.9: 1 which may correspond to a generally higher male population in our country. There are various risk factors for the AAS mentioned in the literature, namely, diabetes mellitus, hypertension, obesity, smoking, alcohol abuse, pneumonia, and malignancies. The majority of AAS patients do not have any comorbidities [5-6]. In our patients, hypertension and diabetes mellitus were the most common comorbid conditions. A large percentage of our patients had a history of smoking. Lindquist et al. also mentioned in their case series that smoking was one of the risk factors for supraglottitis [7]. Forty percent of our patients had a history of drinking cold liquids which led to symptoms of supraglottitis. The ingestion of corrosive and hot drinks are also known to be the risk factors for supraglottitis [8].

The majority of our patients presented with fever, sore throat, dysphagia, dysphonia, and leucocytosis. Most of the literature also describes these symptoms and signs to be frequent presentations [3-4, 9]. The percentage of patients presenting with stridor was 86%, which is much higher than that described in the literature. 

In our study, the thumb sign was positive in the majority of patients whenever a lateral neck soft tissue neck x-ray was done. The thumb sign is due to inflammation and swelling of the epiglottis. Although a fiberoptic nasopharyngeal endoscopy is the most reliable and accurate diagnostic tool for supraglottitis, the thumb sign provides a non-invasive diagnosis with a sensitivity of 88% [10]. Recently, Fujiwara et al. published a meta-analysis with the result that the thumb sign has a specificity of 89.2% and sensitivity of 92.2% [11].

In our study, a CT scan of the neck was done in 44% of the patients. A CT scan is not needed for the diagnosis of supraglottitis [12]. However, it helps to diagnose complications that include an extension of the infection into the neck and chest or formation of multiple abscesses in the oropharyngeal area [3].

In the past, *H. influenzae* was a common microorganism causing supraglottitis. As the immunization against *H. influenzae* was widespread and effective, the frequency of supraglottitis caused by other organisms is increasing. Nowadays, it is caused commonly by *Streptococcus*, *Staphylococcus*, and less frequently in immunosuppressed patients, by *K. pneumoniae*, *E. coli*, *Bacteroides*, anaerobes, *Neisseria*, MRSA, and fungi [3-4, 13]. The *Streptococcus* species were the most common bacteria to cause supraglottitis in our study as well, and only one patient had supraglottitis due to MRSA. Young et al., however, reported that an increasing incidence of AAS is being caused by MRSA [14].

In our patients, the common antimicrobial agent used was ceftriaxone, followed by piperacillin + tazobactam and amoxicillin + clavulanic acid, empirically (as in the majority of our patients, microbiological cultures were not taken) and after microbiological culture and sensitivities were available. The literature also suggests starting empirical treatment with third-generation cephalosporins in these patients [1, 3, 9]. Wu et al. found that third-generation cephalosporins and amoxicillin + clavulanic acid were the most frequently used antimicrobial agents in upper respiratory tract infections, including supraglottitis [15]. Adrenaline nebulization was given to 66% of our patients. It is mentioned that adrenaline nebulization helps by decreasing local tissue edema [3, 16].

All of our patients received steroids as adjuvant therapy due to the anti-inflammatory effect coupled with the stabilization of endothelial permeability, thus, decreasing tissue edema [17]. Recent literature suggests steroids are increasingly used in the treatment of AAS [3, 9, 13, 16].

Fifty percent of our patients did not require any airway interventions, while 39% needed intubation. All of these intubations were difficult and required fiberoptic or video laryngoscopy. Ten percent of our patients required tracheostomy, as endotracheal intubation was impossible. Frantz et al. reported an intubation rate of 9% in supraglottitis patients, as their patients were not managed in the ICU and had milder infections [18]. In a recent study, Tapiovaara et al. found 50% of AAS patients required intubations [19].

In our patients, 12.7% required tracheostomy to secure the airway. Ng et al. reported that only 0.9% of their AAS patients required tracheostomy [4]. Tapiovaara et al. reported that half of their patients required tracheostomy, whereas, in a recent meta-analysis and systematic review of AAS, Sideris et al. found that 10% of patients required airway interventions [19-20].

In our study, a majority of patients recovered without any complications. The most common complication (33%) was the extension of supraglottic infection and abscess formation in the oropharyngeal, sublingual, and submental regions, which is known as Ludwig’s angina. Sixteen percent of our patients had complete respiratory obstruction resulting in apnea.

Our patients who developed Ludwig’s angina presented with fever, dysphagia, or dyspnea and required significantly higher airway interventions, as well as a significantly longer duration of symptoms and a longer stay in the ICU.

The weakness of our study is that it is a single-center, retrospective study and the majority of the patients did not have microbiological investigations. A higher sample size can also increase the power of the study.

## Conclusions

AAS is a potentially life-threatening respiratory infection. In our study, the patients presenting with symptoms had higher rates of stridor. Risk factors included smoking and drinking chilled liquids. The thumb sign was helpful in diagnosis and CT of the neck was required to diagnose the complications. Although in the majority of patients a microbiological workup was not done, frequent microorganisms causing AAS were the *Streptococcus* species. Third-generation cephalosporins, in combination with steroids and adrenaline nebulization, were the predominant medical therapy. Critical complications included apnea and Ludwig’s angina. Patients with a longer duration of symptoms had a higher chance of developing Ludwig’s angina.

## References

[1] Navaratnam AV, Smith ME, Majeed A, McFerran DJ (2015). Supraglottitis: a potential airway emergency that can present in primary care. Br J Gen Pract.

[2] Lichtor JL, Roche Rodriguez M, Aaronson NL, Spock T, Goodman TR, Baum ED (2016). Epiglottitis: it hasn't gone away. Anesthesiology.

[3] Bizaki AJ, Numminen J, Vasama JP, Laranne J, Rautiainen M (2011). Acute supraglottitis in adults in Finland: review and analysis of 308 cases. Laryngoscope.

[4] Ng HL, Sin LM, Li MF, Que TL, Anandaciva S (2008). Acute epiglottitis in adult: a retrospective review of 106 patients in Hong Kong. Emerg Med J.

[5] Suzuki S, Yasunaga H, Matsui H, Fushimi K, Yamasoba T (2015). Factors associated with severe epiglottitis in adults: analysis of a Japanese inpatient database. Laryngoscope.

[6] Abdallah C (2012). Acute epiglottitis: trends, diagnosis and management. Saudi J Anaesth.

[7] Lindquist B, Zachariah S, Kulkarni A (2017). Adult epiglottitis: a case series. Perm J.

[8] Verhees V, Ketharanathan N, Oen IMMH, Baartmans MGA, Koopman JSHA (2018). Beware of thermal epiglottis! A case report describing 'teapot syndrome'. BMC Anesthesiol.

[9] Cruz MGY, Almazan NA (2016). Adult acute epiglottitis: an eight-year experience in a Philippine Tertiary Government Hospital. Philipp J Otolaryngol Head Neck Surg.

[10] Takata M, Fujikawa T, Goto R (2016). Thumb sign: acute epiglottitis. BMJ Case Rep.

[11] Fujiwara T, Miyata T, Tokumasu H, Gemba H, Fukuoka T (2016). Diagnostic accuracy of radiographs for detecting supraglottitis: a systematic review and meta‐analysis. Acute Med Surg.

[12] Ramalanjaona G, Shpak M (2014). Challenges in diagnosis of adult epiglottitis: limitation of CT scan. Emerg Med Open J.

[13] Ramlatchan SR, Kramer N, Ganti L (2018). Back to basics: a case of adult supraglottitis. Cureus.

[14] Young LS, Price CS (2007). Complicated adult epiglottitis due to methicillin resistance staphylococcus aureus. Am J Otolaryngol.

[15] Wu Y, Yang C, Xi H, Zhang Y, Zhou Z, Hu Y (2016). Prescription of antibacterial agents for acute upper respiratory tract infections in Beijing, 2010-2012. Eur J Clin Pharmacol.

[16] Rabeea M, Al Ansari H, Al Abdulla A (2019). An atypical cause of an epiglottis abscess. Case Rep Infect Dis.

[17] Phillips JS, Innes AJ, Naik MS (2004). Corticosteroids for supraglottitis. Br J Anaesth.

[18] Frantz TD, Rasgon BM, Quesenberry CP Jr (1994). Acute epiglottitis in adults. Analysis of 129 cases. JAMA.

[19] Tapiovaara LK, Aro KLS, Bäck LJJ, Koskinen AIM (2019). Comparison of intubation and tracheotomy in adult patients with acute epiglottitis or supraglottitis. Eur Arch Otorhinolaryngol.

[20] Sideris A, Holmes TR, Cumming B, Havas T (2020). A systematic review and meta-analysis of predictors of airway intervention in adult epiglottitis. Laryngoscope.

